# The use of spirometry to define airflow obstruction and diagnose COPD

**DOI:** 10.1183/13993003.00966-2026

**Published:** 2026-08-06

**Authors:** Peter Burney, Ben Knox-Brown, André Amaral

**Affiliations:** 1 National Heart and Lung Institute, Imperial College London, London, UK; 2 Royal Papworth Hospital NHS Foundation Trust, Cambridge, UK

## Abstract

*To the Editor*:

## Abstract 2

**The recent statement from GOLD/GLI on the use of spirometry is insufficient, particularly in the global South** https://bit.ly/4nx13qw

*To the Editor*:

Halpin *et al*. [1], with their viewpoint entitled “Joint statement from GOLD/GLI regarding the use of spirometry to define airflow obstruction and diagnose COPD”, are to be congratulated on getting the Global Lung Function Initiative (GLI) and the Global Initiative for Chronic Obstructive Lung Disease (GOLD) initiative to agree, even if their principal disagreement appears unresolved.

The difference between the “lower limit of normal” (LLN) and the “fixed ratio” for assessing obstruction is that the LLN is adjusted for age, sex and height. The apparent advantage of the fixed ratio is the simplicity of its calculation. With almost all spirometers now containing software to calculate the LLN, the fixed ratio no longer makes sense. What was the point of a GOLD/GLI statement if this disagreement could not now be resolved?

The 1-s forced expiratory volume (FEV_1_)/forced vital capacity (FVC) ratio does not vary substantially between geographic areas (or ethnicities) so the “problem” of ethnic adjustments does not arise when assessing obstruction, and any standard will serve as well – the GLI global equations included.

A more important problem is that most COPD is diagnosed from symptoms and if airflow is measured at all, it is often measured as FEV_1_ or even peak flow. As dyspnoea is as strongly associated with a low FVC as with a low FEV_1_/FVC ratio [2], this leads to overdiagnosis of COPD and underdiagnosis of airflow restriction. This is particularly relevant in low income regions.

We agree with Halpin *et al*. [1] on the need for more spirometry. However, this will not solve the problem of misdiagnosis unless doctors and other health professionals are more aware of the existence and importance of a non-specific low lung volume. This condition is associated with cardiometabolic disorders and reduced survival, and is particularly common in the global South [3].

The question of whether to use FEV_1_ or FVC to assess “severity” is more important than the debate over whether to use the LLN or a fixed ratio to define obstruction. FEV_1_ has been used traditionally, is a good marker of prognosis, and is easier to measure accurately than FVC. However, it is widely misunderstood as assessing severity of obstruction. This is better assessed as the z-score of the FEV_1_/FVC ratio [4]. FVC is even better associated with survival (total lung capacity even more so), but is more difficult to assess accurately. A low FVC is common in the global South (Caribbean, sub-Saharan Africa, and South, Southeast and East Asia) and in African-Americans and migrants from these areas living in the West [5]. As a low FVC is associated with vascular and metabolic disorders [6] and reduced survival [3], it is an important diagnosis in its own right, with or without obstruction.

When assessing FVC (or FEV_1_ as its proxy) the choice of standards is more complicated than when assessing the FEV_1_/FVC ratio:

1) When assessing prognosis, a universal standard should be used [7], but the GLI global equations [8] are an irrational average of an unsystematic sample with high variability where the 95th centile is of little practical value. In assessing this measure, it makes more sense to take an aspirational “normal” value from the USA or Europe. This has the advantages of not underestimating the prevalence of a “low FVC” in Europe and America, and of judging the whole world by the same standard.
2) On rare occasions, when using spirometry to aid in the diagnosis of a specific restrictive disorder, no global value is of any help. These cannot distinguish the low lung volumes associated with specific conditions from the non-specific low lung volumes commonly found in much of the world. Regional values may be helpful, as supplied by the Burden of Obstructive Lung Disease (BOLD) study [9], but it should be noted that even within regions there is considerable variation and going back to local standards may yet be appropriate for this purpose in some places.
